# Mapping Research Knowledge on Rice Husk Ash Application in Concrete: A Scientometric Review

**DOI:** 10.3390/ma15103431

**Published:** 2022-05-10

**Authors:** Muhammad Nasir Amin, Waqas Ahmad, Kaffayatullah Khan, Mohamed Mahmoud Sayed

**Affiliations:** 1 Department of Civil and Environmental Engineering, College of Engineering, King Faisal University, Al-Ahsa 31982, Saudi Arabia; kkhan@kfu.edu.sa; 2 Department of Civil Engineering, COMSATS University Islamabad, Abbottabad 22060, Pakistan; waqasahmad@cuiatd.edu.pk; 3 Architectural Department, Faculty of Engineering and Technology, Future University in Egypt, New Cairo 11845, Egypt; mohamed.mahmoud@fue.edu.eg

**Keywords:** rice husk ash, concrete, supplementary cementitious material, waste management, scientometric analysis, eco-friendly construction material

## Abstract

This study aimed to carry out a scientometric review of rice husk ash (RHA) concrete to assess the various aspects of the literature. Conventional review studies have limitations in terms of their capacity to connect disparate portions of the literature in a comprehensive and accurate manner. Science mapping, co-occurrence, and co-citation are a few of the most difficult phases of advanced research. The sources with the most articles, co-occurrences of keywords, the most prolific authors in terms of publications and citations, and areas actively involved in RHA concrete research are identified during the analysis. The Scopus database was used to extract bibliometric data for 917 publications that were then analyzed using the VOSviewer (version: 1.6.17) application. This study will benefit academics in establishing joint ventures and sharing innovative ideas and strategies because of the statistical and graphical representation of contributing authors and countries.

## 1. Introduction

Increased greenhouse gas (GHG) discharges have caused the melting of the Antarctic and Arctic polar ice caps. This has resulted in significant environmental problems on Earth [1]. The manufacture and transportation of building materials, as well as the installation and construction of structures, require considerable energy and produce significant volumes of GHG. In the European Union’s member states, buildings use around 50% of the total energy consumption and contribute to almost 50% of the CO_2_ emissions in the environment over their life cycle, which includes construction, operation, and destruction [2,3]. The building sector is still experiencing an increase in demand for concrete [4,5,6,7,8,9]. Ordinary Portland cement (OPC) is a critical component of concrete that contributes considerably to GHG emissions [10,11,12,13]. OPC production causes around 5–8% of worldwide CO_2_ emissions [14,15,16,17]. Annual cement usage is over 4000 million tons and is predicted to reach approximately 6000 million tons by 2060 [18]. These GHG emissions have been a significant contributor to climate change [19,20,21]. In recent years, there has been a rise in the figure of thorough studies on the many triggers of climate change (natural and man-made), their effects on living conditions, and possible adaptation and mitigation techniques [22,23,24,25,26,27]. Blended cement manufacturing demands the use of a number of different cementitious components because of the higher energy and emission issues associated with OPC production [28]. Industrial waste utilization as supplementary cementitious materials (SCMs) is one of the methods that might cause a significant reduction in the usage of OPC, while also eliminating the risks connected with the disposal of waste materials from varied sectors [29,30,31,32,33]. Therefore, the most efficient technique for reducing the carbon footprint of the construction industry is to replace OPC with suitable alternative SCMs [34,35,36,37]. There are several binders that might be utilized in concrete to decrease GHG emissions from the concrete industry [38,39]. Utilizing recycled/waste materials in concrete is a viable method of mitigating the impact of environmental challenges [40]. This not only meets the increasing need for concrete, but also significantly reduces the direct danger to society [41]. Numerous researchers in the building sector have focused on the utilization of waste resources, particularly SCMs [42,43]. The production of environmentally friendly concrete has been critical to decreasing GHG emissions [44]. Agriculture wastes such as rick husk ash (RHA), sugarcane bagasse ash, olive oil ash, etc., as well as industrial wastes, are being utilized to partially replace OPC in the manufacture of sustainable concrete [45,46,47,48,49]. By polluting air and water systems, dumping these waste materials in the open ground creates a major environmental threat [50]. Globally, rice husk is produced by nearly 110 million tons and RHA by 22 million tons [51]. Rice husk is effectively and extensively used as a fuel in numerous nations for rice paddy milling operations and electricity production facilities [52]. This procedure results in the formation of a pozzolanic substance known as RHA, which contains more than 75% silica by weight (after incineration, 20% of the rice husk remains in the form of RHA) [23]. The ash formed by this operation is often dumped into water flows, contaminating the water and causing ecological damage [53]. Utilizing waste materials in concrete might enhance the durability and strength of the material owing to the pozzolanic effect [54]. This decreases industrial demand for OPC, lowering the expense of producing concrete and mitigating the negative impacts of CO_2_ discharges during the OPC production process [28]. Given RHA’s advantageous characteristics as an SCM, its use is not limited to cementitious concrete, but may also include geopolymer concrete, self-compacting concrete, fiber-reinforced concrete, pavement blocks, bricks, and high-performance nanocomposites [55,56,57,58,59,60,61].

**Figure 1:**
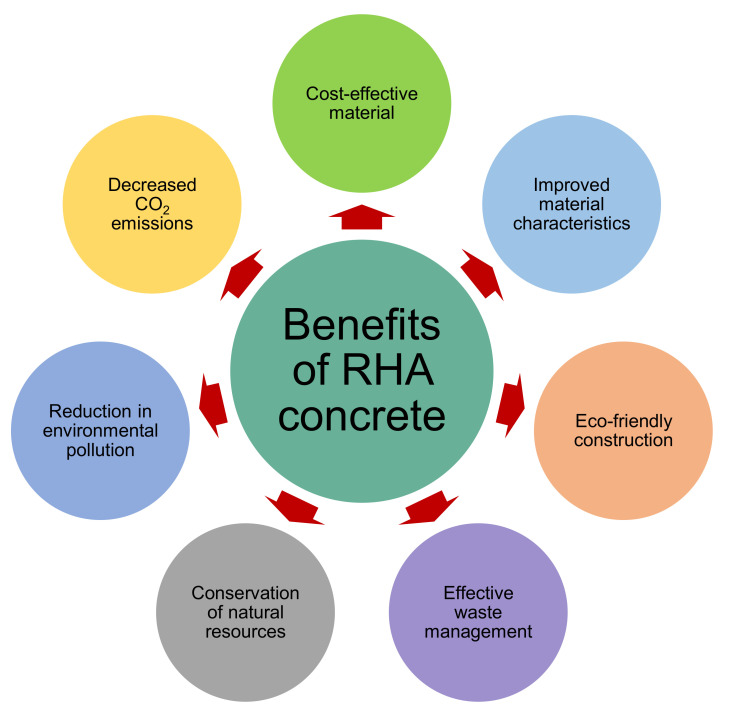
Benefits of RHA concrete.

The key properties of SCMs are their compatibility with aggregates (similar to OPC) and their better pozzolanic nature [62,63]. The application of RHA in concrete has sparked tremendous interest in the usage of sustainable and environmentally friendly SCM [64,65,66,67]. RHA has amorphous nature, high surface area, and compatibility with OPC-concrete, which results in outstanding pozzolanic capabilities [55,68,69,70]. Each kilogram of rice milled yields 0.28 kg of rice husk [71]. As a result, an enormous quantity of waste is generated annually. These rice husks are utilized as a fuel source in a variety of sectors to generate heat energy, including incineration and combustion units [72,73,74]. After the complete burning of rice husk, around 20–25% RHA by weight is formed [56]. A very small quantity of the RHA is subsequently employed as a field fertilizer, and sadly, most of it is thrown in open landfills [73,75]. RHA includes amorphous silica and calcium oxide and so may be utilized efficiently as an SCM in concrete [76,77,78]. Utilizing RHA in concrete results in better durability and strength, reduced material expenditures owing to OPC savings, and ecological advantages associated with waste material disposal [48]. RHA has been employed in recent studies as a partial replacement for OPC as well as fine aggregate in concrete mixes [79,80,81]. The properties of RHA concrete vary by the amount of OPC or fine aggregate replaced, the RHA grain size, the chemical characteristics of RHA independent of the water-cement ratio, and aggregate size/shape in the matrix [82]. However, for optimum strength growth, it is advised that around 10–25% of OPC be replaced [55,56]. The use of RHA in concrete has a number of benefits, as depicted in Figure 1. RHA has been researched for its possible use in cement-based composites as SCM or fine aggregate replacement. Also, natural aggregate extraction uses substantial energy and leads to increased CO_2_ discharges [83]. As a result, issues about the manufacturing and use of OPC may be reduced, while natural resources can be conserved. Thus, including RHA into cementitious materials reduces the demand for OPC and fine aggregate and results in an ecologically beneficial building material. Furthermore, waste management issues can be alleviated by the use of RHA in construction materials.

As research on RHA concrete develops in response to the expanding environmental concerns, scientists face information constraints that may stymie creative investigation and scholarly collaboration. As a result, it is vital to create and apply a method that enables researchers to obtain critical information from the most reliable sources feasible. A scientometric method may assist in overcoming this shortcoming via the software application. The intention of this work is to conduct a scientometric analysis of bibliographic records published on RHA concrete up to 2021. Using a proper software tool, a scientometric analysis may undertake a quantitative examination of massive bibliometric data. Conventional review studies are weak in their ability to connect diverse sections of the literature in a complete and accurate manner. Science mapping, co-occurrence, and co-citation are a few of the most demanding parts of modern-day exploration [84,85,86]. The scientometric analysis identifies sources with the most articles, keyword co-occurrence, the most prolific authors in terms of papers and citations, and areas actively engaged in RHA concrete research. The Scopus database was utilized to extract bibliometric data for 917 relevant articles, which were then evaluated using the VOSviewer program. As a consequence of the statistical and graphical depiction of authors and countries, this study will aid academics in forming joint ventures and exchanging novel ideas and methods.

## 2. Methods

For the quantitative evaluation of the various features of the bibliographic data, this study carried out the scientometric analysis of the bibliographic data [87,88,89]. Numerous papers have been written on the issue, and it is critical to use a search engine that is reputable. Scopus and Web of Science are two very accurate search engines that are particularly well-suited for this purpose [90,91]. The bibliographic data for this study on RHA concrete were gathered using Scopus, which comes highly recommended by academics [92,93]. As of March 2022, a Scopus search for “rice husk ash concrete” found 1234 articles. Numerous filter preferences were employed to eradicate superfluous documents. The document types “journal article”, “conference paper”, “journal review”, and “conference review” were selected. “Journal” and “conference proceeding” were chosen as the “source type”. The “publication year” restriction was set to “2021”, and the “language” constraint was set to “English”. For further examination, the “subject areas” of “engineering”, “material science”, and “environmental science” were selected. A total of 917 records were kept following the application of these requirements. Numerous researchers have likewise reported on the same technique [94,95,96].

**Figure 2:**
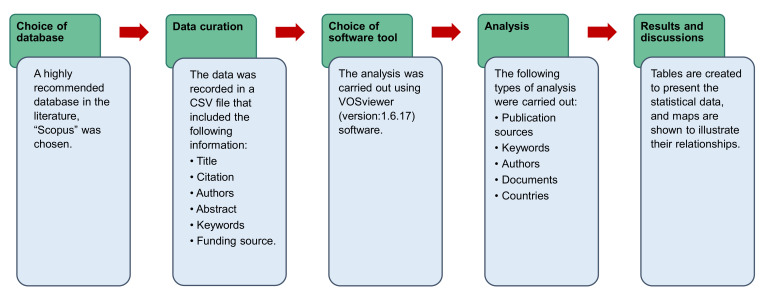
Sequence of the research methods.

Scientometric investigations employ scientific mapping, a technique developed by academics for the purpose of analyzing bibliometric information [97]. Scopus records were saved in the Comma Separated Values (CSV) (see Supplementary Materials) files for further evaluation using appropriate computer software. VOSviewer (version: 1.6.17) was employed to generate the scientific visualization and quantitative assessment of the literature from the retrieved records. VOSviewer is an easily available and open-source mapping tool that is broadly employed across a range of areas and is well-suggested by academics [98,99,100,101]. As a result, the current study’s goals were satisfied through the use of the VOSviewer. The obtained CSV files were loaded into the VOSviewer, and additional assessment was performed while retaining data integrity and consistency. During the bibliographic assessment, the sources of publications, the highly regularly appearing keywords, the scholars with the most publications and citations, and the country’s participation were all assessed. The many facets, their relationships, and co-occurrence were shown graphically, while their statistical figures were reported in tables. The flowchart of the scientometric strategy is depicted in Figure 2.

## 3. Analysis of Results

### 3.1. Relevant Subject Areas and Yearly Publications

**Figure 3:**
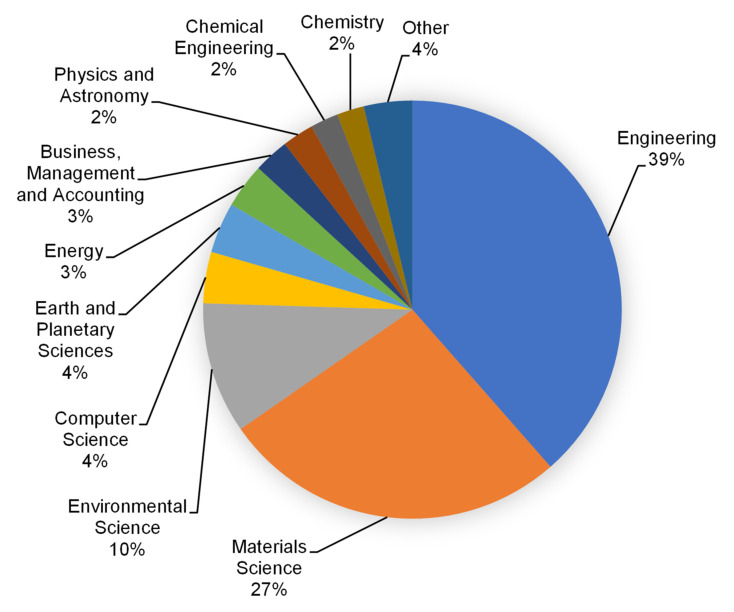
The subject area of articles.

**Figure 4:**
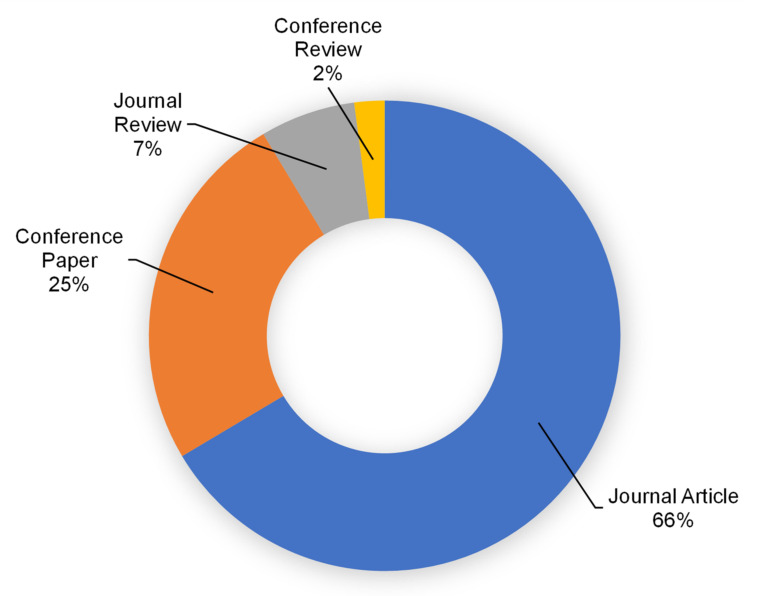
Various types of documents published in the related study field.

**Figure 5:**
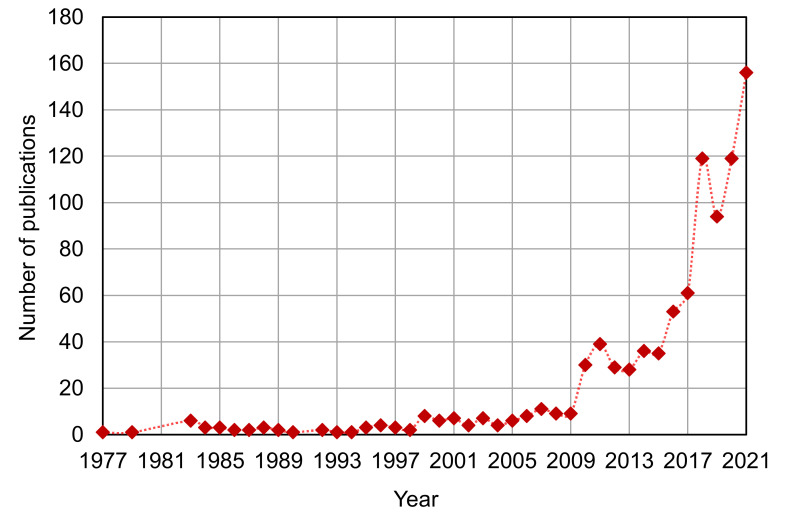
Annual publication trend of articles.

The Scopus analyzer was employed to carry out this analysis to discover the most pertinent study fields. Engineering, materials science, and environmental science were found to be the leading three document-producing areas, with around 39, 27, and 10% of documents, respectively, accounting for a total 76% of contributions based on document count, as seen in Figure 3. Additionally, as seen in Figure 4, the kind of paper was evaluated in the searched term in the Scopus database. According to this research, journal articles, conference papers, journal reviews, and conference reviews accounted for almost 66, 25, 7, and 2% of total documents, respectively. The yearly trend in publications in the present research area from 1977 to 2021 is depicted in Figure 5, since the first document on the subject research field was discovered in 1977. In the research of RHA concrete, a slow increase in the amount of publications was seen, with an average of roughly three papers per year up to 2000. Following this, there was a continuous increase in publications, with an average of roughly 20 papers each year from 2001 to 2016. The quantity of publications increased significantly during the previous five years (2017–2021), averaging approximately 110 each year.

### 3.2. Sources of Publications

The assessment of publication sources was carried out using the VOSviewer on the collected bibliographic data. During the analysis, “bibliographic coupling” was selected as the “kind of analysis”, while “sources” were retained as the “unit of analysis”. At least ten papers per source restraint were set, and 14 of the 265 publication sources met these criteria. Table 1 shows the publishing sources that published a minimum of ten documents, providing data on RHA concrete, up to 2021, together with the amount of citations obtained during that time period. The main three sources/journals based on paper count are “Construction and building materials”, “IOP conference series: materials science and engineering”, and “Materials today: proceedings”, with 110, 48, and 45 papers, respectively. Moreover, the top three sources based on the overall citations are “Construction and building materials” with 6797, “Cement and concrete composites” with 2268, and “Journal of cleaner production” with 1579. Remarkably, this exploration would provide a basis for upcoming scientometric investigations in the research of RHA concrete. In addition, prior traditional reviews were unable to generate scientific visualization maps.

**Figure 6:**
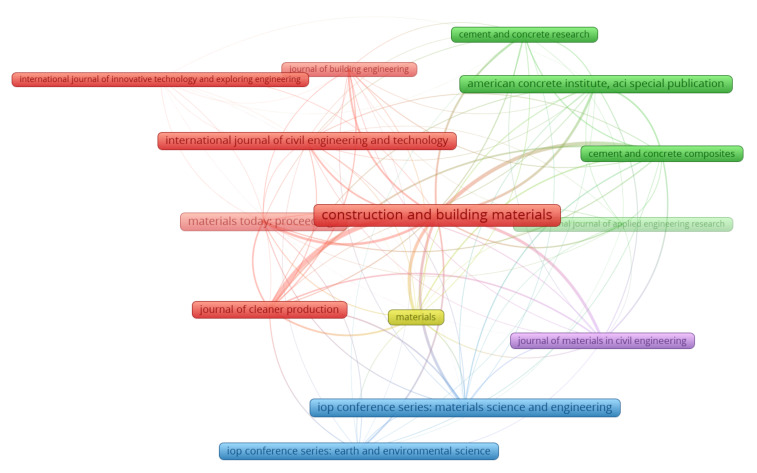
Scientific visualization of publication sources with at least ten publications in the related research area.

Figure 6 illustrates a map of journals that have published a minimum of ten documents. The box size is proportional to the journal’s impact on the current research area’s document quantity; a bigger box dimension implies a superior impact. As an example, “Construction and building materials” has a bigger box than the others, implying that it is a source of considerable importance in that field. Five clusters were created, each of which is represented in the artwork by a different hue (red, blue, green, yellow, and purple). Clusters are formed on the basis of the research source’s extent or the frequency with which they are co-cited in a similar article [102]. The VOSviewer created clusters of journals based on their co-citation patterns in published papers. For instance, the red cluster consists of six sources that have been co-cited several times in identical works. Additionally, nearly spaced frames (journals) in a cluster have stronger relationships than widely distributed frames. For instance, “Construction and building materials” is more strongly correlated with “Materials today: proceedings” than with “Journal of cleaner production”.

### 3.3. Keywords

**Figure 7:**
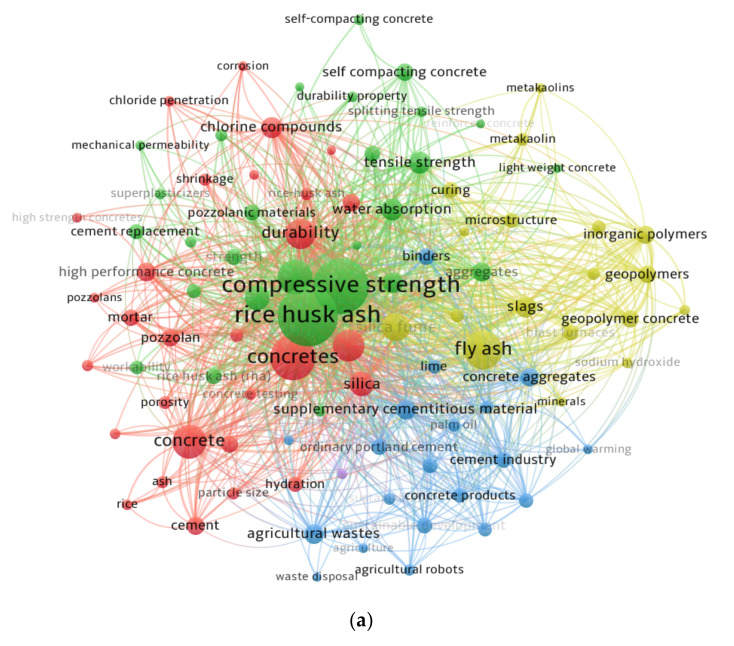

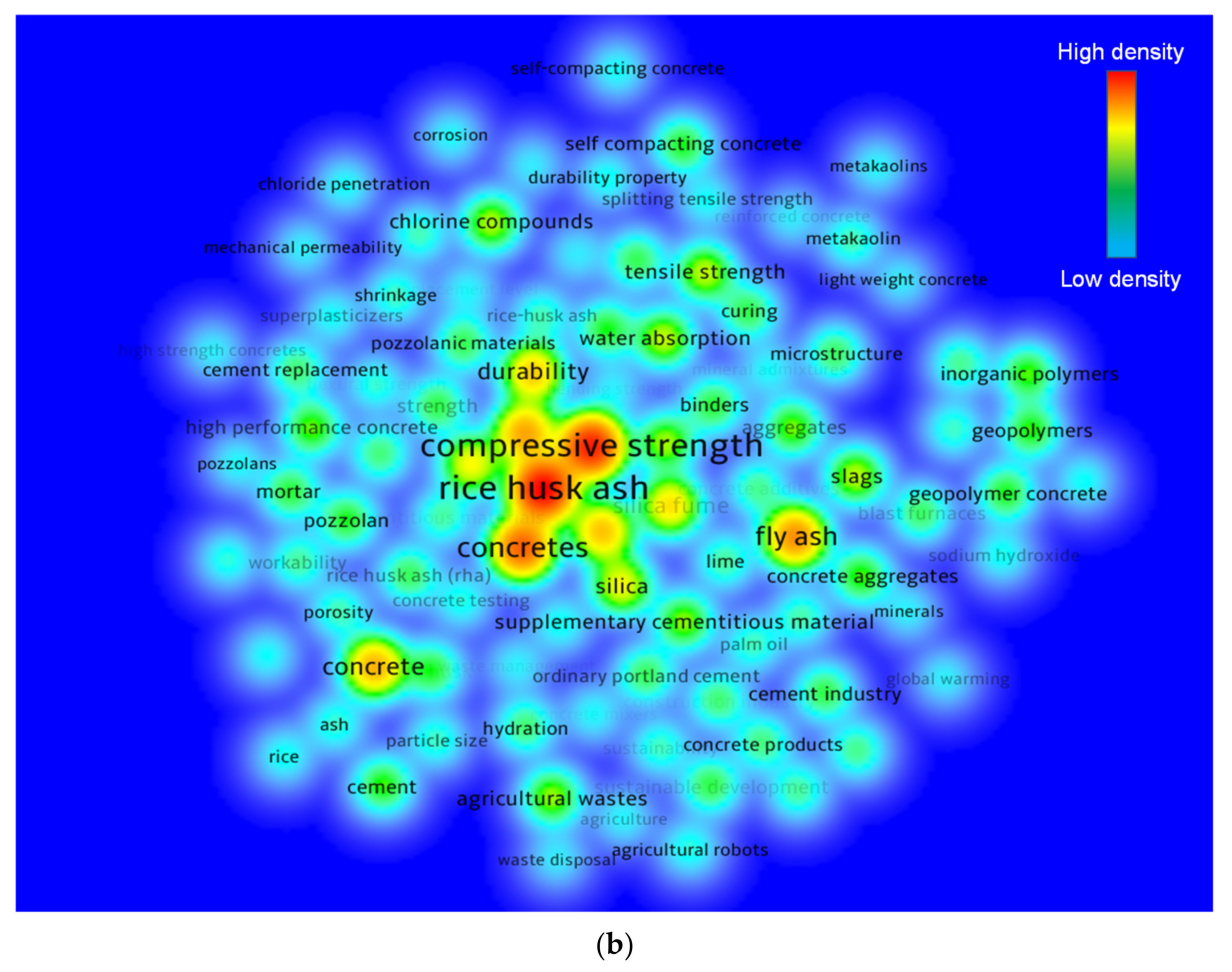
Keywords analysis: (**a**) scientific visualization; (**b**) density visualization.

Keywords are important in research because they define and highlight the study domain’s fundamental subject [103]. The “analysis type” was set to “co-occurrence” and the “analysis unit” to “all keywords” for the evaluation. The least repetition constraint for a keyword was maintained at 20, and 96 of the 4185 keywords were retained. The leading 20 keywords most commonly used in published articles in the topic area are listed in Table 2. Rice husk ash, compressive strength, concretes, fly ash, and cements are the five most-often appearing keywords in the subject research area. According to the keyword analysis, RHA has been studied primarily as an SCM in normal concrete, self-compacting concrete, and high-performance concrete, as well as a precursor material in geopolymers. Figure 7 depicts a visualization map of keywords in terms of co-occurrences, linkages, and the density related to their frequency of occurrence. In Figure 7a, the size of a keyword circle implies its frequency, whereas its position implies its co-occurrence in articles. Also, the graph illustrates that the leading keywords have wider circles than the others, implying that they are critical terms for RHA concrete research. Clusters of keywords have been highlighted in the graph in a way that reflects their co-occurrence across a range of publications. The color-coded clustering is based on the co-occurrence of numerous keywords in published publications. The existence of four clusters is indicated by distinct colors (blue, red, green, and yellow) (Figure 7a). As seen in Figure 7b, different colors indicate varying concentrations of keyword density. The colors red, yellow, green, and blue are organized, corresponding to their density concentrations, with red indicating the highest and blue indicating the lowest density concentration. Compressive strength, rice husk ash, and concretes all exhibit red signs implying a higher concentration of density. This discovery will assist aspiring authors in choosing keywords that will facilitate the identification of published data in a certain field.

### 3.4. Authors

**Figure 8:**
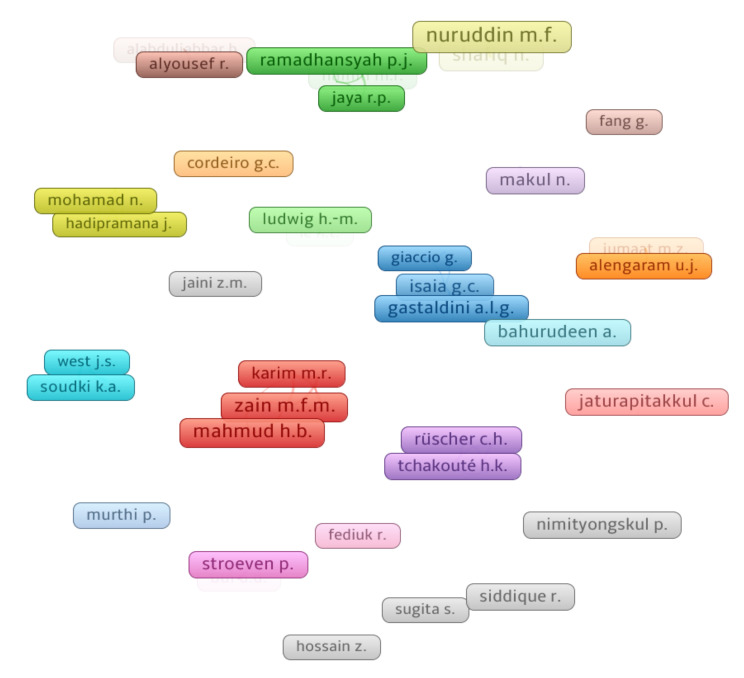
Scientific visualization of authors that published articles in the related research area.

Citations indicate a researcher’s influence within a certain study domain [104]. For the evaluation of authors, the “kind of analysis” was chosen “co-authorship”, and the “unit of analysis” was chosen “authors”. The minimal paper restrictions for a writer were kept at 5, and 50 of the 2226 authors met this condition. Table 3 summarizes the most prolific authors in terms of publications and citations in the research of RHA concrete, as determined by data obtained from the Scopus search engine. The average citations for each author were calculated by dividing the total citations by the total publications. It will be difficult to quantify a scientist’s efficacy when all factors such as the number of publications, total citations, and average citations are included. In contrast, the writer’s assessment will be determined independently of each factor, i.e., total publications, total citations, and average citations. Nuruddin M.F. is the leading author with 16, followed by Zain M.F.M. and Mahmud H.B. with 14 each, and Shafiq N. with 13 publications. Jaturapitakkul C. leads the field in terms of total citations with 973, Zain M.F.M. is second with 738, and Chindaprasirt P. is third with 668 total citations in the current study area. Furthermore, when comparing average citations, the following writers stand out: Jaturapitakkul C. has around 97, Chindaprasirt P. has approximately 84, and Bui D.D. has approximately 82 average citations. Figure 8 illustrates the relationship between authors who have published at least ten publications and the most eminent authors. It was noticed that the largest set of connected authors based on citations are 6 of the 60 authors. This study revealed that a small number of writers are connected by citations in the research of RHA concrete.

### 3.5. Documents

**Figure 9:**
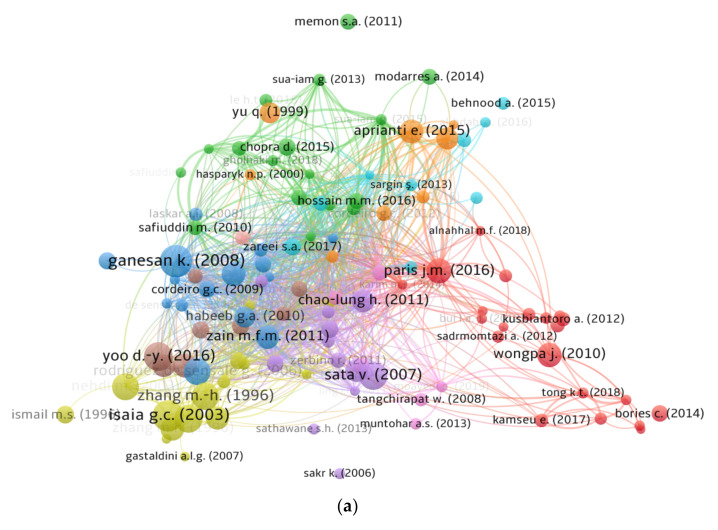

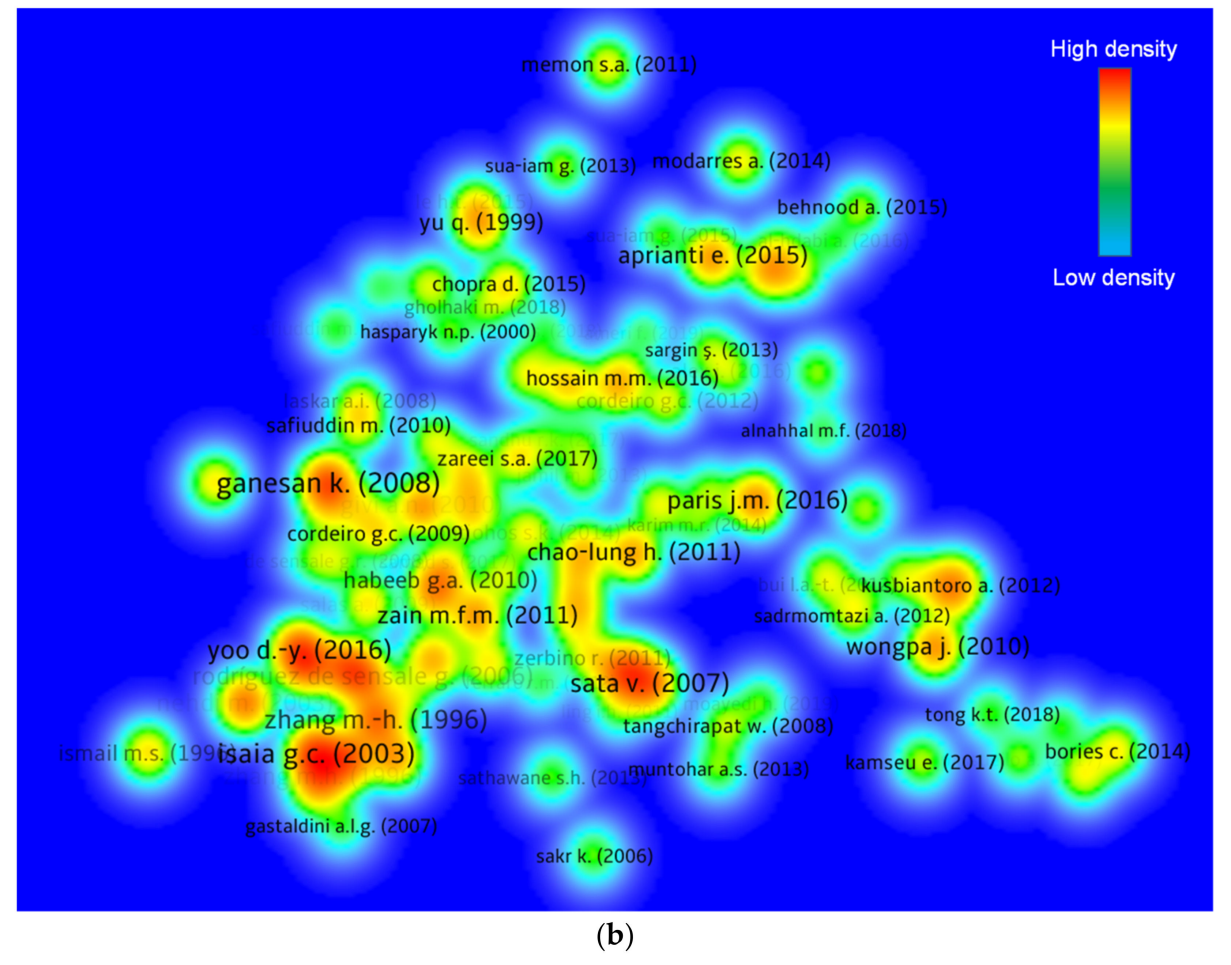
Scientific mapping of published articles in the related research area up to 2021; (**a**) connected articles in terms of citations, (**b**) density of connected articles.

The amount of citations a document obtains reflects its influence on a certain area of research. Papers with a high citation count are recognized as pioneers in their respective fields of research. For the assessment of documents, the “kind of analysis” was set to “bibliographic coupling” and “unit of analysis” to “documents”. The least citations requirement for a document was 50, and 121 of 917 documents satisfied this requirement. The top ten papers in the area of RHA concrete by citations are included in Table 4, along with their writers and citation information. Ganesan K. [105] received 346 citations for their article “Rice husk ash blended cement: Assessment of optimal level of replacement for strength and permeability properties of concrete”. G.C. Isaia [106] and D.-Y. Yoo [107] received 329 and 228 citations, respectively, for their publications and were positioned in the leading three. However, up until 2021, only 18 publications received more than 200 citations. In addition, Figure 9 illustrates the map of linked papers based on citations, as well as the density of those documents in the current study subject. The analysis revealed that 112 of 121 papers were linked by citations. Figure 9a illustrates the citation-based mapping of connected articles. Also, the density mapping (Figure 9b) reveals the top articles’ enhanced density concentration.

### 3.6. Countries

**Figure 10:**
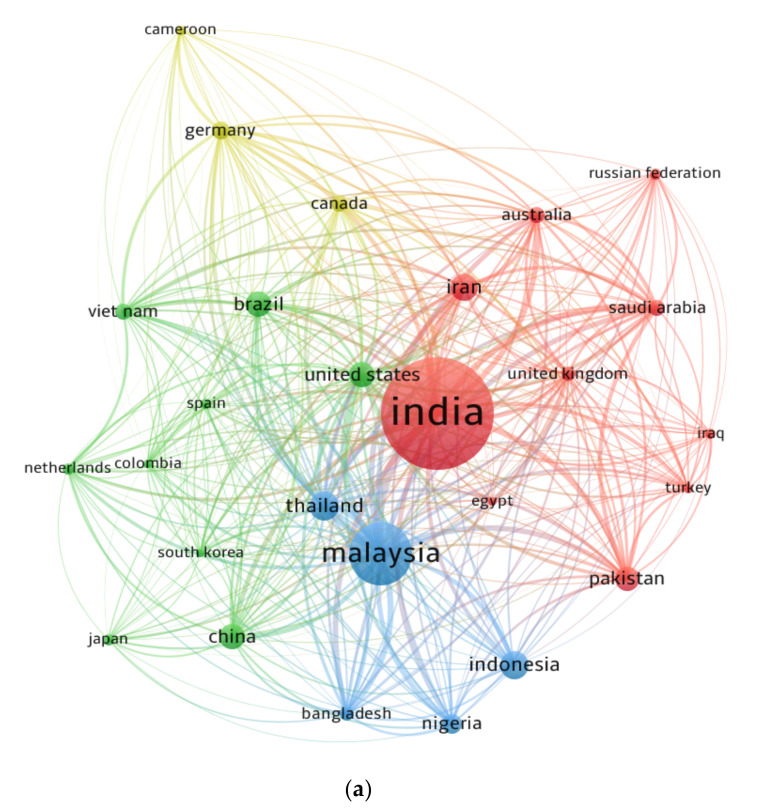

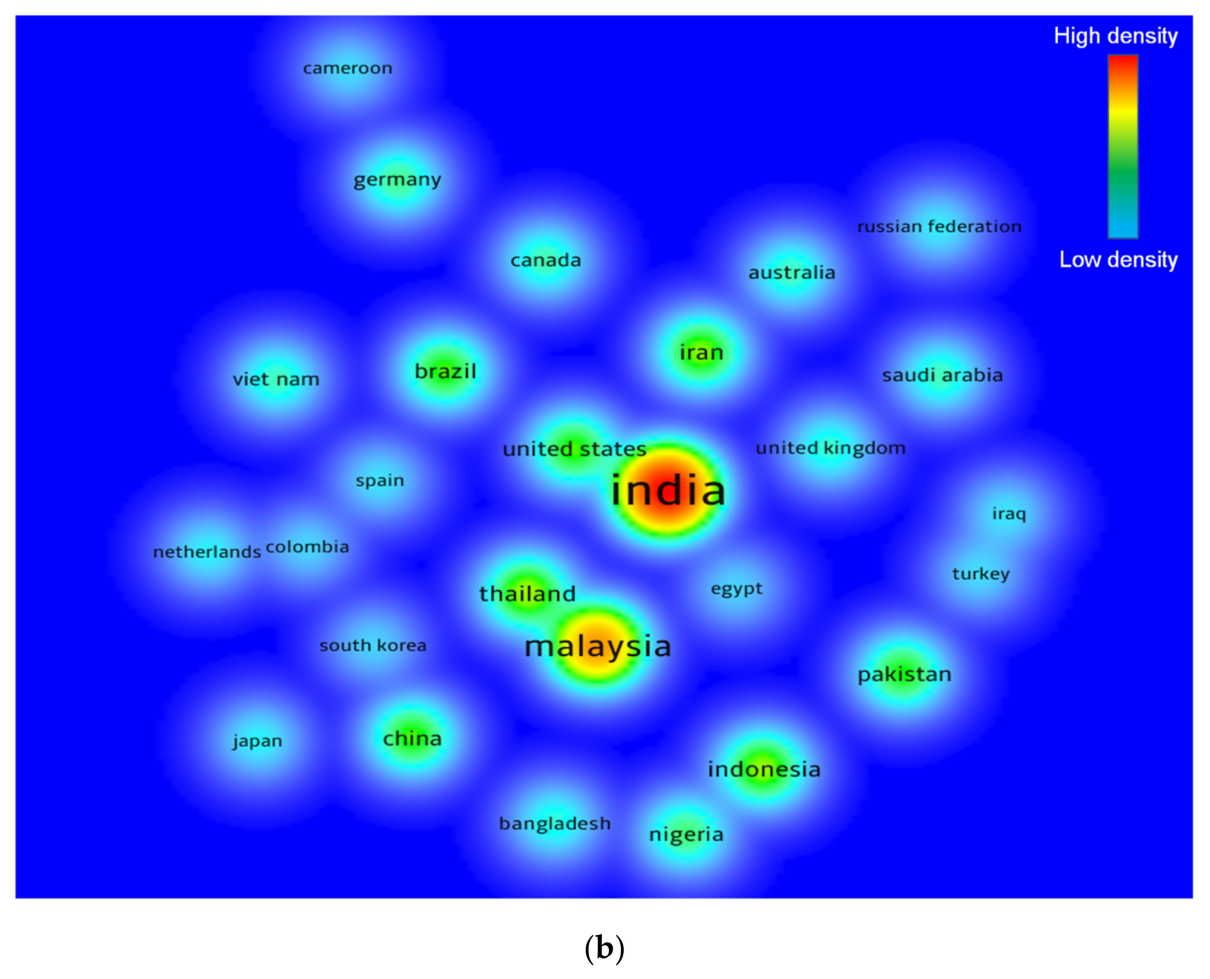
Scientific visualization countries with at least ten publications in the related research area up to 2021: (**a**) network visualization; (**b**) density visualization.

Several countries have contributed more to current research than others have and are expected to contribute further. The network map was created to allow readers to view areas committed to the research of RHA concrete. “Bibliographic coupling” was selected as the “kind of analysis”, and “countries” as the “unit of analysis”. The minimum document limit for a nation was set at 10, and 27 countries met this requirement. The nations listed in Table 5 have published at least ten documents in the present study field. India, Malaysia, and Thailand presented the most papers with 293, 133, and 48 documents. Moreover, these nations received the most citations, with Malaysia receiving 3104, India receiving 3098, and Thailand receiving 2049 citations. Figure 10 illustrates the visualization of the science mapping as well as the density of nations connected via citations. The size of a box is proportional to a nation’s effect on the subject research (Figure 10a). The nations with the most engagement had a higher density, as indicated by the density visualization (Figure 10b). The statistical and graphical analysis of the contributing states will aid emerging researchers in establishing scientific alliances, forming joint ventures, and exchanging innovative techniques and ideas. Researchers from nations interested in promoting research on RHA concrete can work with experts in the field and profit from their experience.

## 4. Discussions and Future Perspectives

This study provided a statistical overview and mapping of various aspects of the literature on RHA concrete. Previous manual review studies have limitations in terms of their ability to comprehensively and accurately connect diverse sections of the literature. This study identified sources (journals) that published most articles, most commonly employed keywords in the published papers, articles and authors having most citations, and countries actively involved in the research of RHA concrete. The analysis of keywords identified that RHA had been examined for its possible applications as SCM in conventional concrete, self-compacting concrete, and high-performance concrete due to the presence of high silica content in its chemical composition [114,115,116,117,118]. In addition, the use of RHA is also researched for manufacturing geopolymer concrete [119,120,121]. RHA provides several advantages when used in concrete. RHA has been investigated for prospective use as a cement or fine aggregate substitute in concrete. The issues associated with manufacturing and the use of cement might be decreased [122]. Also, because natural aggregate extraction consumes a significant amount of energy and results in higher CO_2_ emissions [123]. As a consequence, concerns regarding natural resource depletion may be alleviated. Thus, the incorporation of RHA into concrete minimizes the need for cement and fine aggregate, resulting in a more environmentally friendly construction material [124]. By incorporating RHA into construction materials, waste management difficulties can be solved [73]. In addition, the most active and contributing countries in terms of publications were identified from the literature and their connections based on citations. The statistical and graphical representations of the contributing states will assist developing scholars in creating scientific partnerships, establishing joint ventures, and exchanging novel approaches and ideas. Researchers from countries interested in advancing RHA concrete research can collaborate with professionals in the area and benefit from their knowledge.

Most of the RHA applications stated above are still in their development, and more in-depth analyses are necessary before broadening their applicability [71]. Furthermore, in the present practice, the utilization of RHA concrete in full-scale reinforced concrete structures under service and high loading circumstances has not been examined. Additionally, there are currently no clear standards for the preparation, processing, and use of RHA on a larger scale. In the available literature, researchers have solely relied on their intuition to determine the optimal degree of cement and fine aggregate replacement using RHA [105]. Additionally, previous work has not explored the compatibility and long-term durability of RHA concrete. Steel reinforcement corrosion in RHA-blended concrete must be researched in water, chloride, sulphate, and acidic environments over an extended period of time. Also, because information on the life cycle evaluation of RHA concrete is limited and needs to be thoroughly examined. To enhance the strength of concrete, alternative and supplemental additives such as nano-silica and fibers can be added to RHA concrete. Additionally, the high concentration and coarser character of RHA allow for the formation of a porous and less dense matrix of the concrete. Nonetheless, the addition of nano-clay, short fibers, and nano-silica to concrete has demonstrated the ability to increase its density, shock resistance, and tensile stress resistance. As a result, these additives combined with RHA-blended concrete may provide another sustainable material for future construction.

## 5. Conclusions

The objective of this study was to conduct a scientometric analysis of the available literature on rice husk ash (RHA) concrete in order to assess various measures. The Scopus database was queried for 917 relevant papers, and the results were analyzed using the VOSviewer program. The following findings were drawn from this study:

- An analysis of publication sources containing documents on RHA concrete research exposed that the topmost three sources are “Construction and building materials”, “IOP conference series: materials science and engineering”, and “Materials today: proceedings”, with 110, 48, and 45 papers, respectively, Also, the leading three publication sources in terms of overall citations are “Construction and building materials” with 6797, “Cement and concrete composites” with 2268, and “Journal of cleaner production” with 1579.
- A keyword analysis of the subject study field shows that the five most-often appearing keywords are rice husk ash, compressive strength, concretes, fly ash, and cements. The keyword analysis revealed that RHA had been studied primarily as a supplemental cementitious material (SCM) in concrete.
- Author analysis revealed that only 50 writers had published at least five publications on RHA concrete research. The top writers were classified according to their number of publications, citations, and average citations. Nuruddin M.F., with 16, Zain M.F.M., and Mahmud H.B., with 14 each, and Shafiq N., with 13 papers, are the top three authors in terms of overall publications. With 973 citations, Jaturapitakkul C. leads the field, followed by Zain M.F.M. with 738 and Chindaprasirt P. with 668 citations until 2021. In addition, when the average number of citations is compared, the following authors stand out: C. Jaturapitakkul has around 97, P. Chindaprasirt has approximately 84, and D.D. Bui has approximately 82 average citations.
- According to an analysis of papers providing data on RHA concrete, Ganesan K. [105] received 346 citations for their article “Rice husk ash blended cement: Assessment of optimal level of replacement for strength and permeability properties of concrete”. G.C. Isaia [106] and D.-Y. Yoo [107] received 329 and 228 citations, respectively, for their publications and were positioned in the best three. Moreover, only 18 publications acquired more than 200 citations in the subject area from 2011 to 2021.
- The leading nations were assessed based on their participation in RHA concrete research, and it was determined that only 27 countries published at least ten papers. India, Malaysia, and Thailand each delivered 293, 133, and 48 papers, respectively. In addition, these nations received the most citations, with Malaysia receiving 3104, India receiving 3098, and Thailand receiving 2049 citations.
- RHA has been investigated for its potential uses as SCM in conventional concrete, self-compacting concrete, and high-performance concrete because of the high silica concentration in its chemical composition. Furthermore, RHA is being investigated for application in the production of geopolymer concrete.
- The application of RHA in the construction sector will result in green construction by reducing cement demand and conserving natural sources when used as a substitute for cement and fine aggregate.
- The majority of the RHA applications are still under investigation, and further analysis is required before widening their effectiveness.

## Figures and Tables

**Table 1:** Publication sources with at least ten publications in the related research field up to 2021.

S/N	Publication Source	Number of Publications	Total Number of Citations
1	Construction and building materials	110	6797
2	IOP conference series: materials science and engineering	48	110
3	Materials today: proceedings	45	227
4	American concrete institute, ACI special publication	35	261
5	International journal of civil engineering and technology	32	54
6	IOP conference series: earth and environmental science	28	22
7	Journal of cleaner production	27	1579
8	Cement and concrete composites	21	2268
9	Journal of materials in civil engineering	17	424
10	Cement and concrete research	14	1539
11	materials	13	215
12	Journal of building engineering	10	144
13	International journal of applied engineering research	10	96
14	International journal of innovative technology and exploring engineering	10	10

**Table 2:** The leading 20 frequently employed keywords in the research of RHA concrete.

S/N	Keyword	Occurrences
1	Rice husk ash	460
2	Compressive strength	402
3	Concretes	284
4	Fly ash	214
5	Cements	185
6	Concrete	173
7	Portland cement	154
8	Durability	146
9	Silica fume	126
10	Mechanical properties	106
11	Silica	103
12	Tensile strength	83
13	Water absorption	83
14	Slags	81
15	Chlorine compounds	78
16	Concrete mixtures	75
17	Agricultural wastes	72
18	Supplementary cementitious material	70
19	Aggregates	66
20	High performance concrete	64

**Table 3:** Authors with at least five publications in the research of RHA concrete up to 2021.

S/N	Author	Number of Publications	Total Number of Citations	Average Citations
1	Nuruddin M.F.	16	309	19
2	Zain M.F.M.	14	738	53
3	Mahmud H.B.	14	397	28
4	Shafiq N.	13	260	20
5	Jaturapitakkul C.	10	973	97
6	Makul N.	10	335	34
7	Isaia G.C.	9	635	71
8	Gastaldini A.L.G.	9	581	65
9	Stroeven P.	9	357	40
10	Rüscher C.H.	9	196	22
11	Ramadhansyah P.J.	9	90	10
12	Bahurudeen A.	9	56	6
13	Chindaprasirt P.	8	668	84
14	Siddique R.	8	487	61
15	Karim M.R.	8	380	48
16	Sua-Iam G.	8	334	42
17	Tchakouté H.K.	8	189	24
18	Nimityongskul P.	8	59	7
19	Jamil M.	7	441	63
20	Alengaram U.J.	7	222	32
21	Hainin M.R.	7	44	6
22	Mohamad N.	7	31	4
23	Bui D.D.	6	492	82
24	Ludwig H.-M.	6	437	73
25	Cordeiro G.C.	6	290	48
26	Soudki K.A.	6	248	41
27	Kamseu E.	6	214	36
28	Leonelli C.	6	214	36
29	Raman S.N.	6	213	36
30	Alyousef R.	6	73	12
31	Jaya R.P.	6	68	11
32	Murthi P.	6	24	4
33	Islam M.N.	5	327	65
34	Le H.T.	5	308	62
35	Safiuddin M.	5	247	49
36	West J.S.	5	247	49
37	Giaccio G.	5	229	46
38	Zerbino R.	5	229	46
39	Sugita S.	5	209	42
40	Jumaat M.Z.	5	167	33
41	Gobinath R.	5	64	13
42	Wan Ibrahim M.H.	5	60	12
43	Alabduljabbar H.	5	40	8
44	Fediuk R.	5	39	8
45	Hossain Z.	5	39	8
46	Samad A.A.A.	5	28	6
47	Jaini Z.M.	5	22	4
48	Hadipramana J.	5	10	2
49	Riza F.V.	5	10	2
50	Fang G.	5	0	0

**Table 4:** The top ten highly cited published articles up to 2021 in the research of RHA concrete.

S/N	Article	Title	Total Number of Citations Received
1	Ganesan K. [105]	Rice husk ash blended cement: Assessment of optimal level of replacement for strength and permeability properties of concrete	346
2	Isaia G.C. [106]	Physical and pozzolanic action of mineral additions on the mechanical strength of high-performance concrete	329
3	Yoo D.-Y. [107]	Mechanical properties of ultra-high-performance fiber-reinforced concrete: A review	288
4	Bui D.D. [108]	Particle size effect on the strength of rice husk ash blended gap-graded Portland cement concrete	283
5	Sata V. [109]	Influence of pozzolan from various by-product materials on mechanical properties of high-strength concrete	278
6	Zhang M.-H. [110]	High-performance concrete incorporating rice husk ash as a supplementary cementing material	271
7	Rodríguez De Sensale G. [53]	Strength development of concrete with rice-husk ash	261
8	Nehdi M. [111]	Performance of rice husk ash produced using a new technology as a mineral admixture in concrete	257
9	Paris J.M. [112]	A review of waste products utilized as supplements to Portland cement in concrete	232
10	Wongpa J. [113]	Compressive strength, modulus of elasticity, and water permeability of inorganic polymer concrete	223

**Table 5:** Leading countries based on published documents in the present research area until 2021.

S/N	Country	Number of Publications	Total Number of Citations
1	India	293	3098
2	Malaysia	133	3104
3	Thailand	48	2049
4	Indonesia	46	271
5	Iran	44	1528
6	Brazil	39	1294
7	United States	39	1180
8	China	39	818
9	Pakistan	37	728
10	Nigeria	28	360
11	Germany	25	729
12	Canada	22	1270
13	Vietnam	21	1334
14	Australia	21	459
15	Saudi Arabia	21	225
16	United Kingdom	19	394
17	Bangladesh	18	481
18	Netherlands	14	834
19	Japan	14	450
20	Russian Federation	13	186
21	Iraq	12	225
22	Spain	12	153
23	Turkey	11	358
24	Cameroon	11	295
25	South Korea	10	736
26	Egypt	10	240
27	Colombia	10	223

## Data Availability

The data used in this research have been properly cited and reported in the main text.

## Supplementary Materials

The following supporting information can be downloaded at: https://www.mdpi.com/article/10.3390/ma15103431/s1. Table S1: Data retrieved from the Scopus database and used for the analysis.
